# Risk of osteoporotic fractures in menopausal women with common mental health diagnoses prescribed SSRIs/SNRIs: cohort and self-controlled case series analyses

**DOI:** 10.1007/s11657-024-01459-3

**Published:** 2024-10-23

**Authors:** Dana Alsugeir, Matthew Adesuyan, Christina Avgerinou, Vikram Talaulikar, Li Wei, Ruth Brauer

**Affiliations:** 1 Research Department of Practice and Policy, UCL School of Pharmacy, BMA House, Tavistock Square, London, WC1H 9JP UK; 2 Pharmacy Practice Department, College of Clinical Pharmacy, Imam Abdulrahman Bin Faisal University, Dammam, Saudi Arabia; 3 Department of Primary Care and Population Health, University College London, London, UK; 4 Reproductive Medicine Unit, University College London Hospital, London, UK

**Keywords:** Fractures, Osteoporosis, SSRIs/SNRIs, Menopausal

## Abstract

***Summary*:**

In a population-based cohort study of menopausal women with common mental health diagnoses, SSRIs/SNRIs were associated with a 32% increased risk of osteoporotic fractures. The risk of osteoporotic fractures was particularly increased for longer periods of treatment with SSRIs/SNRIs (> 5 years) and in younger menopausal women (< 50 years old).

**Purpose:**

To investigate the association between selective serotonin reuptake inhibitors (SSRIs) and serotonin-norepinephrine reuptake inhibitors (SNRIs) and the risk of osteoporotic fractures (OF) in menopausal women with common mental health diagnoses (CMHD).

**Methods:**

We conducted the study with two designs (cohort and self-controlled case series [SCCS]), using the IQVIA Medical Research Database (IMRD) UK. The source population comprised women aged ≥ 50 years and women with a record indicating menopause (< 50 years). All women had a recorded CMHD. For the cohort analysis, the risk of OFs was estimated by comparing women prescribed SSRIs/SNRIs (exposed) to those not exposed. Cox regression was used to estimate hazard ratios (HR) with 95% confidence intervals (CIs). For the SCCS, women acted as their own controls; periods of exposure to SSRIs/SNRIs were compared to periods of non-exposure using conditional Poisson regression to estimate incidence rate ratios (IRR) with 95% CIs.

**Results:**

We identified 292,848 women, of whom 35,222 experienced OFs within a median follow-up of 6.01 years. We found strong evidence of an association between SSRIs/SNRIs and the risk of OFs (adjusted HR = 1.32, 95% CI:1.29–1.35). Compared to periods of no exposure, SSRIs/SNRIs increased the risk of OFs during the first 30 days (IRR = 1.38, 95% CI:1.26–1.51), during the first 90 days (IRR = 1.58, 95% CI: 1.48–1.69), and the remaining exposure (IRR = 1.42, 95% CI:1.37–1.48).

**Conclusions:**

In a population of menopausal women with CMHDs, the prescribing of SSRIs/SNRIs antidepressants was associated with a higher risk of OFs. Careful assessment of osteoporosis risk needs to be considered when treating menopausal women with SSRIs/SNRIs antidepressants.

## Introduction

Menopause, the end of the menstrual cycle, is characterised by symptoms associated with the loss of the protective effects of oestrogen [1]. Vasomotor symptoms, such as hot flushes, are well-known direct effects of the loss of oestrogen [2]. Indirectly, the risk of various morbidities increases, including the risk of osteoporosis [2]. Postmenopausal women have a two-fold increased risk of new osteoporotic fractures (OF) compared to men [3, 4]. Moreover, during the peri-menopausal and postmenopausal period, women are at higher risk of common mental health diagnoses (CMHDs) [5]. Various mental health disorders, as well as pharmacological treatment options for CMHD, are independently associated with an increased risk of osteoporotic fractures [6, 7]. In particular, the use of selective serotonin reuptake inhibitors (SSRIs) and serotonin-norepinephrine reuptake inhibitors (SNRIs) is associated with an increased risk of OFs [8]. SSRIs/SNRIs have been shown to inhibit bone formation by increasing serotonin levels peripherally [9, 10]. A recent meta-analysis found that SSRIs were associated with an increased risk of fractures in the general population [11]. A study conducted in perimenopausal women without CMHD found a 60% increased risk of OFs in users of SSRIs [12].

The aim of this study was to investigate the additive risk of osteoporotic fractures in menopausal women with CMHDs, prescribed SSRI/SNRIs in the UK in a within-person and between-person analysis.

## Methods

### Data source

A population-based study with cohort and self-controlled case series designs was conducted using data from the IQVIA Medical Research Database (IMRD), incorporating data from THIN, a Cegedim database [13]. Reference made to THIN is intended to be descriptive of the data asset licensed by IQVIA. This database contains pseudonymised electronic primary care data from over 16 million patients, representing approximately 6% of the UK population [14]. UK primary care clinical diagnoses and prescribing data were extracted from the IMRD-UK database for the duration of the study period (1 January 2000 to 2 November 2021). UK primary care data have been validated for the study of osteoporotic fractures [3].

### Study population

We defined our source population as (1) women with a primary care record indicating menopause before their 50th birthday, or, if no record of menopause before the age of 50, (2) women were assumed to be menopausal when they turn 50 years old to allow for the inclusion of women without a recorded diagnosis of menopause (82%) [15]. The term menopausal women is used to describe perimenopausal and postmenopausal women. All women were required to have at least one recording of CMHDs after entry into the study cohort, regardless of any past diagnoses. Common mental health diagnoses, as per NICE guidelines, were defined as depression, generalised anxiety disorder, panic disorder, obsessive–compulsive disorder (OCD), post-traumatic stress disorder (PTSD), and social anxiety disorder [16]. Women were excluded from the study population if they had the outcome of interest (osteoporotic fracture) before the study entry.

### Exposure

The study population was divided based on exposure status during the study period. Initiation of SSRIs/SNRIs was determined by prescription records, using drug code lists. Please refer to Table 5 in the Appendix for code lists of study medications. New users comprised women who received two or more consecutive prescriptions (within 180 days) for SSRIs/SNRIs. A 180-day washout period was used to distinguish new users from continuous users. Given the absence of dispensing data in the source database, women with a single prescription of SSRIs/SNRIs were not considered users and were excluded from the cohort. The comparison group consisted of menopausal women with CMHDs and without a recorded prescription for SSRIs/SNRIs. The index date for the treatment group was the date of the second prescription [17]. For the control group, the index date was randomly assigned based on the distribution of index dates of the exposed group using incidence density sampling [18]. To minimise immortal time bias, follow-up started after eligibility and treatment assignment. We censored follow-up at the earliest of the following: OFs, death, disenrollment, or end of the study period (2 November 2021).

### Outcome

Bone mineral density measurements are not recorded in the IMRD-UK database. Therefore, osteoporotic fractures were assumed based on the anatomical site where the fracture occurred, as recorded in patients’ records. The primary outcome was the diagnosis of osteoporotic fractures measured using READ codes, adapted from the code list provided by Khalid et al. [19]. The code list excluded fractures of the face, skull, and digits as they are unlikely to be OFs. Only the first recorded fracture after the start of follow-up was defined as an outcome event. Please refer to the OF code list in Table 6 in the Appendix.

### Covariates

We adjusted for risk factors for OF and potential confounders associated with exposure to SSRIs/SNRIs and OF; all were measured before or at the index date. At index date, we measured age and calendar year, Townsend deprivation index (five quintiles), body mass index (BMI) (underweight: < 18.59 kg/m^2^; healthy weight: 18.6–24.9 kg/m^2^; overweight: 25–29.9 kg/m^2^; obese: 30–39.9 kg/m^2^; severely obese: ≥ 40 kg/m^2^), alcohol status (non-drinker, average drinker, problematic drinker, drinker of unknown quantity, and ex-drinker), and smoking status (ex-smoker, never smoked, and current smoker). Analyses were also adjusted for comorbidities predisposing to secondary osteoporosis: hypogonadism (premature menopause, bilateral oophorectomy or orchidectomy, anorexia nervosa, chemotherapy for breast cancer, hypopituitarism), inflammatory bowel diseases, diseases causing prolonged immobility, rheumatoid arthritis, organ transplantation, diabetes, thyroid disorder, chronic obstructive pulmonary disease, and heart failure. We also controlled for the following comorbidities as binary variables: history of CMHDs before menopause, severe mental illness, and recorded diagnosis of osteoporosis. History of falls and fractures (excluded from the outcome) were controlled for if ever occurred before the index date. The following medications were adjusted for if prescribed during the 180 days before the index date: other psychotropic medications, MHT, calcium, and vitamin D, prescriptions for osteoporosis treatment (bisphosphonates, denosumab, teriparatide, calcitonin, raloxifene, other selective oestrogen receptor modulators, tibolone, parathyroid hormone, and strontium ranelate), and corticosteroids. Lastly, parity (as a binary variable) was included in the analyses [20].

### Statistical analysis

Descriptive statistics were carried out using percentages for binary variables. Median and interquartile ranges (IQR) or mean and standard deviation (SD) were used for continuous variables. The association between the prescribing of SSRIs/SNRIs and the risk of OFs was investigated by comparing women prescribed SSRIs/SNRIs to those not prescribed using the Cox proportional hazard regression model with adjusted hazard ratios (aHRs) and 95% confidence intervals (CI). Visual inspection of the Kaplan–Meier survival graph was used to check the proportional hazard assumption. Multiple imputation with chained equations was used to impute the missingness in the variables of smoking, alcohol, BMI, and Townsend deprivation quintiles. The number of imputations used was 12, based on the largest percentage of missing data [21]. Hazard ratios were adjusted to address potential confounding bias due to non-randomisation of SSRIs/SNRIs treatment initiation, and propensity scores (PS) were estimated for each patient at baseline, using a logistic regression model conditional on the covariates listed above. The PS is defined as the conditional probability of receiving the treatment of interest (SSRIs/SNRIs), based on a given set of baseline characteristics [22] and was further used to calculate the inverse probability of treatment weighting (IPTW) [19]. IPTW was used in addition to the truncation of extreme weights at the 99th and 1st percentiles [23]. Standardised mean differences (SMD) were used to compare the difference between the exposed and unexposed groups before and after weighting. An SMD of less than 0.1 was considered balanced [24].

To assess the interaction of the following factors with prescribing SSRIs/SNRIs, sub-group analyses were conducted:

1. To test the hypothesis that the use of menopausal hormone therapy (MHT) could mitigate the risk of OFs in women prescribed SSRIs/SNRIs, the study cohort was stratified based on prior exposure to MHT (yes/no). MHT exposure was assessed 180 days before the index date;
2. We performed a subgroup analysis by age (at index date). The age categories were < 50 years, 50–59 years, 60–69 years, 70–79 years, and ≥ 80 years;
3. Women prescribed SSRIs/SNRIs were grouped based on the duration of exposure: less than 1 year, ≥ 1 to < 2 years, ≥ 2 to < 5 years, and ≥ 5 years. For this analysis, we used a grace period of 180 days between two recorded prescriptions;
4. The risk of OFs in women prescribed SSRIs and SNRIs was analysed separately.

To assess the robustness of our primary results, we conducted three sensitivity analyses:

1. To assess the robustness of the assumed age of menopause, we conducted two analyses assuming a different inclusion age at 45 and 55 years;
2. We created a lag period after the index date; for this analysis, the first 6 months of follow-up were excluded for both exposed and unexposed groups [25];
3. Women who received a diagnosis or treatment for osteoporosis and those with a history of fracture before the index date were excluded.

**Fig. 1:**
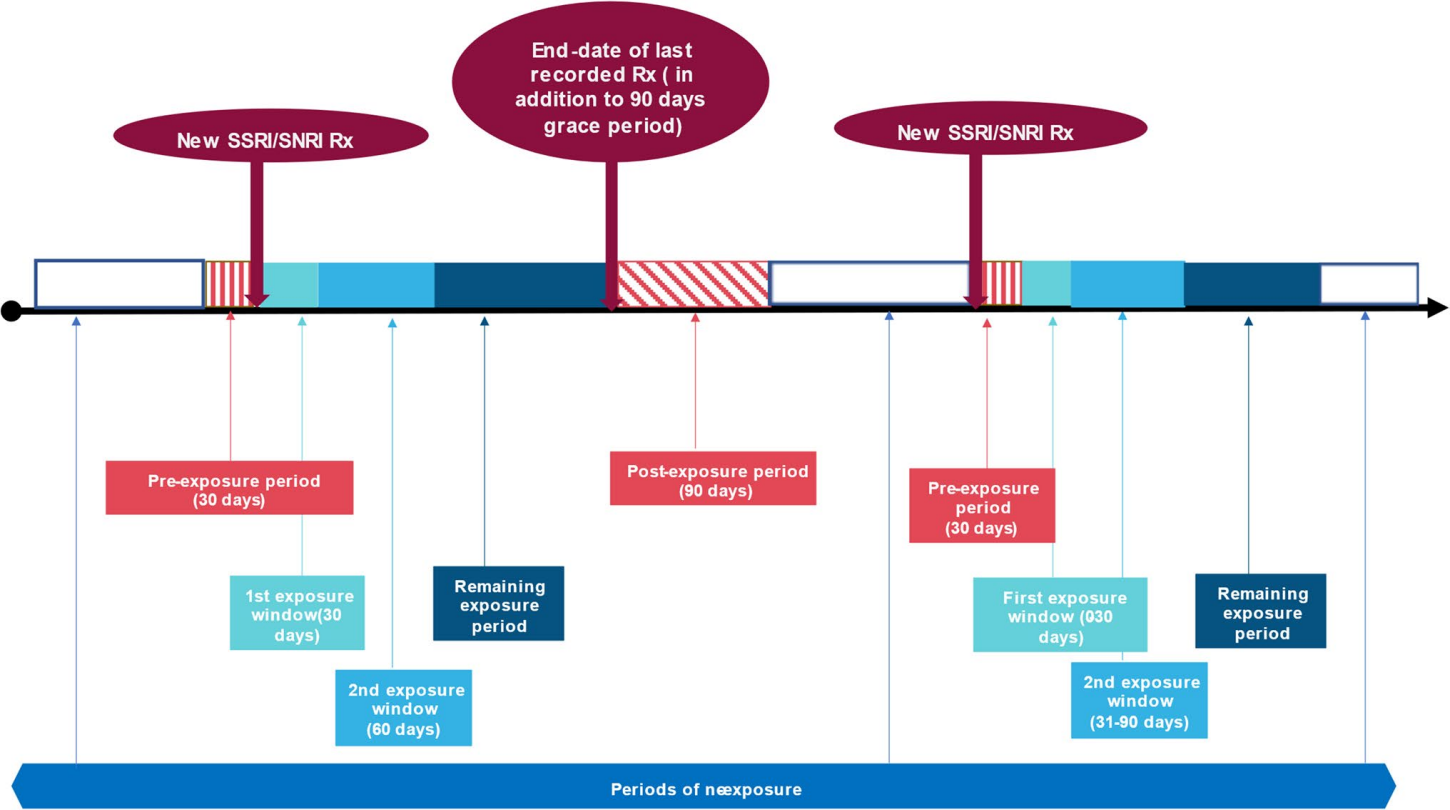
Baseline and risk periods for women prescribed SSRIs/SNRIs compared with periods of no exposure to SSRIs/SNRIs in the self-controlled case series design

A secondary analysis was conducted using a different study design: We used a self-controlled case series (SCCS) analysis to assess the risk of OFs associated with the prescribing of SSRIs/SNRIs. The self-controlled case series allows for within-person comparison and eliminates the effect of any between person-confounders that do not vary over time. The observation time of every woman with a recorded OF and exposure to SSRIs/SNRIs was divided into the following risk windows: (1) 1–30 days before being prescribed SSRIs/SNRIs [26], (2) the first 30 days of exposure, (3) a second exposure window of 60 days (days 31 to 90 of treatment), (4) remaining exposure period, and (5) a post-exposure period of 90 days. Comparison periods or baseline risk periods were defined as periods of no exposure to SSRIs/SNRIs. A graphical illustration of the baseline and risk periods applied in this secondary analysis is shown in Fig. 1. For the statistical analysis of the self-controlled case series, we used conditional Poisson regression, conditioned on the number of OFs events, to estimate incidence rate ratios (IRRs) with 95% CIs. The analysis was adjusted according to the age of women.

## Results

After applying the inclusion criteria, 292,848 menopausal women were included in the cohort study. The median follow-up was 6.01 years (IQR: 2.59–10.54). The mean age of women upon index date was 62.40 (SD: 12.29). The most commonly recorded morbidity was a history of CMHDs and were prescribed medications for hypertension. SSRIs/SNRIs were prescribed to 177,865 women. The baseline characteristics of the study subjects and the SMD before and after weighting using IPTW are shown in Table 1. All measured covariates were well balanced across the exposed and non-exposed groups after IPTW.

**Table 1:** Baseline characteristics of the study cohort

Characteristic		Not prescribed SSRIs/SNRIs	Prescribed SSRIs/SNRIs	SMD* before weighting	SMD after weighting
Number		114,983	177,865		
Age at entry, mean (SD)		61.94 (13.27)	62.70 (11.60)	− 0.10	− 0.01
Age at entry	< 50	21,526 (18.7%)	5833 (3.3%)		
	50–59	31,639 (27.5%)	84,938 (47.8%)		
	60–69	28,092 (24.4%)	39,856 (22.4%)		
	70–79	20,565 (17.9%)	26,538 (14.9%)		
	≥ 80	13,161 (11.4%)	20,700 (11.6%)		
Body mass index	Underweight	3796 (3.3%)	5085 (2.9%)	0.08	0.01
	Healthy weight	42,656 (37.1%)	61,135 (34.4%)		
	Overweight	34,812 (30.3%)	53,612 (30.1%)		
	Obese	23,413 (20.4%)	40,107 (22.5%)		
	Severely obese	3287 (2.9%)	6988 (3.9%)		
	Missing	7019 (6.1%)	10,938 (6.1%)		
Smoking status	Ex-smoker	27,999 (24.4%)	42,723 (24.0%)	0.08	0.00
	Never smoked	66,740 (58.0%)	93,972 (52.8%)		
	Current smoker	19,203 (16.7%)	39,238 (22.1%)		
	Missing	1041 (0.9%)	1932 (1.1%)		
Alcohol intake	Ex-drinker	4018 (3.9%)	7390 (4.5%)	0.03	0.00
	Average drinker	49,345 (48.1%)	76,729 (46.8%)		
	Problematic drinker	9152 (8.9%)	17,459 (10.6%)		
	Drinker-unknown quantity	23,915 (23.3%)	39,943 (24.4%)		
	Non-drinker	16,106 (14.01%)	22,479 (12.64%)		
	Missing	12,447 (10.83%)	13,865 (7.8%)		
Townsend deprivation quintiles	Lowest score	25,183 (21.9%)	36,464 (20.5%)	0.03	0.00
	2	23,650 (20.6%)	34,620 (19.5%)		
	3	21,974 (19.1%)	34,025 (19.1%)		
	4	18,716 (16.3%)	30,620 (17.2%)		
	Highest score	13,072 (11.4%)	21,539 (12.1%)		
	No record	12,388 (10.8%)	20,597 (11.6%)		
History of pregnancy		45,179 (39.3%)	71,897 (40.4%)	0.04	0.00
Osteoporosis		5187 (4.5%)	7521 (4.2%)	− 0.01	0.00
Family history of osteoporosis		190 (0.2%)	294 (0.2%)	0.00	0.00
History of fractures		7009 (6.1%)	7879 (4.4%)	− 0.05	0.00
History of falls		9234 (8.0%)	15,608 (8.8%)	0.03	0.00
Rheumatoid arthritis		1567 (1.4%)	2864 (1.6%)	0.02	0.00
Chronic obstructive pulmonary disease		6898 (6.0%)	12,917 (7.3%)	0.05	0.00
Thyroid disorders		2667 (2.3%)	4354 (2.4%)	0.01	0.00
Heart failure		2626 (2.3%)	4579 (2.6%)	0.02	0.00
Diabetes		11,067 (9.6%)	17,219 (9.7%)	0.01	0.01
Diseases causing prolonged immobility		3130 (2.7%)	6550 (3.7%)	0.06	0.00
Diseases causing hypogonadism**		7090 (6.2%)	16,111 (9.1%)	0.11	0.01
Severe mental illness		1984 (1.7%)	2853 (1.6%)	− 0.01	− 0.01
Irritable bowel disease		1174 (1.0%)	2075 (1.2%)	0.02	0.00
History of common mental health diagnoses		57,412 (49.9%)	86,448 (48.6%)	− 0.01	0.00
Combination of calcium and vitamin D		2746 (2.3%)	9815 (5.5%)	− 0.04	− 0.01
Calcium		2538 (2.2%)	1472 (0.8%)	− 0.06	− 0.03
Vitamin D		1578 (1.3%)	981 (0.5%)	0.00	0.00
Corticosteroids		4208 (3.7%)	10,669 (6.0%)	0.12	0.01
Osteoporosis treatment		4731 (4.1%)	8624 (4.8%)	0.03	0.00
Menopausal hormone therapy (MHT) treatment		1647 (1.4%)	6747 (3.8%)	0.19	0.04
Other antidepressants		6477 (5.63%)	13,968 (7.8%)	− 0.22	− 0.05
Antipsychotics		876 (0.76%)	2747 (1.5%)	0.00	− 0.04
Anxiolytics and hypnotics		8271 (7.19%)	22,139 (12.44%)	0.13	− 0.06
Opioids treatment		13,317 (11.6%)	32,835 (18.5%)	0.18	0.10
Antihypertensive treatment		45,837 (39.9%)	84,772 (47.7%)	0.13	0.09

*SMD standardised mean difference, *SD* standard deviation

**Diseases causing hypogonadism (bilateral oophorectomy or orchidectomy, anorexia nervosa, chemotherapy for breast cancer, hypopituitarism)

**Fig. 2:**
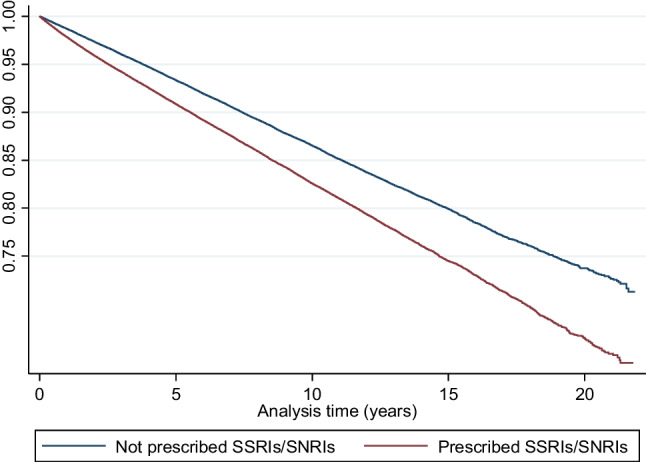
Kaplan–Meier graph for the adjusted primary analysis of the risk of osteoporotic fractures associated with prescribing of SSRIs/SNRIs

During 2,048,923 person-years of follow-up, there were 35,222 individuals with newly diagnosed osteoporotic fractures. The crude incidence rate of OFs in women prescribed SSRIs/SNRIs was 19.49 (95% CI: 19.23–19.76) per 1000 person-years compared with 14.61 (95% CI: 14.37–14.85) per 1000 person-years in women not prescribed SSRIs/SNRIs. Results of the unadjusted Cox regression for the primary outcome of risk of OFs associated with prescribing SSRIs/SNRIs showed a HR of 1.34 (95% CI: 1.31–1.37). After adjusting for confounding variables, we found strong evidence that the initiation of SSRIs/SNRIs in a cohort of women diagnosed with CMHD was associated with an increased risk of OFs, compared to non-users (the adjusted HR = 1.32, 95% CI: 1.29–1.35) (Table 2). The IPTW-adjusted Kaplan–Meier curve for the risk of OFs is shown in Fig. 2.

**Table 2:** Event rates and HR from the primary analysis

Analysis	Events	Person-years 1000	Crude IR (95% CI)* per 1000	Crude HR (95% CI)	Adjusted HR (95% CI)**
Primary outcome: osteoporotic fractures					
Non-user	14,134	967.21	14.61 (14.37–14.85)	*Ref*	*Ref*
SSRIs/SNRIs user	21,088	1081.7	19.49 (19.23–19.76)	1.34 (1.31–1.37)	1.32 (1.29–1.35)

*Per 1000 women-years at risk

**Adjusted using PS-IPTW

The results of subgroup analyses are presented in Table 3. The risk of OFs was not increased in women prescribed MHT along with SSRIs/SNRIs (HR = 0.91, 95% CI: 0.59–1.40). The risk of OFs was highest in women younger than 50 years old (HR = 1.51, 95% CI: 1.36–1.68). The risk increased gradually with increased duration of prescribing. In women with less than 1 year of prescribing, a smaller risk (HR = 1.21, 95% CI: 1.18–1.25) was found, compared to women with 5 or more years of SSRIs/SNRIs prescribing (HR = 1.49, 95% CI: 1.44–1.55). The risk of OFs was slightly higher in women prescribed only SNRIs (HR = 1.36, 95% CI: 1.29–1.44) compared to women prescribed SSRIs (HR = 1.31, 95% CI: 1.28–1.34). The risk of OFs was sustained through several sensitivity analyses (see Table 3).

**Table 3:** Crude and adjusted HRs (SSRIs/SNRIs vs non-SSRIs/SNRIs) for the subgroup and sensitivity cohort analyses

Subgroups	Number of women	Crude HR (95% CI)	Adjusted HR (95% CI)**
MHT status			
Prescribed MHT	8394	0.96 (0.64–1.45)	0.91 (0.59–1.40)
Not prescribed MHT	264,566	1.33 (1.30–1.36)	1.31 (1.28–1.34)
Age groups			
≤ 50 years old	27,359	1.52 (1.38–1.69)	1.51 (1.36–1.68)
50–59 years old	116,577	1.24 (1.19–1.29)	1.24 (1.18–1.29)
60–69 years old	67,948	1.29 (1.28–1.35)	1.28 (1.22–1.33)
70–79 years old	47,103	1.34 (1.28–1.41)	1.31 (1.25–1.37)
≥ 80 years old	33,861	1.37 (1.30–1.44)	1.33 (1.26–1.41)
Duration of use			
< 1 year	207,176	1.23 (1.19–1.26)	1.21 (1.18–1.25)
≥ 1 to < 2 years	144,942	1.31 (1.26–1.36)	1.29 (1.25–1.36)
≥ 2 to < 5 years	147,916	1.56 (1.50–1.62)	1.47 (1.41–1.53)
≥ 5 years	138,558	1.47 (1.42–1.52)	1.49 (1.44–1.55)
Medication class			
SSRIs	280,299	1.33 (1.30–1.36)	1.31 (1.28–1.34)
SNRIs	128,734	1.41 (1.35–1.48)	1.36 (1.29–1.44)
Sensitivity analyses			
Inclusion entry age (> 45)	358,042	1.30 (1.27–1.33)	1.25 (1.22–1.28)
Inclusion entry age (> 55)	275,560	1.28 (1.25–1.30)	1.21 (1.18–1.24)
Introducing a lag period of 180 days	276,317	1.31 (1.28–1.34)	1.31 (1.28–1.35)
Excluding women with osteoporosis, a family history of osteoporosis, and a history of fractures	260,048	1.63 (1.59–1.67)	1.56 (1.52–1.60)

**Adjusted using PS-IPTW

In the secondary SCCS analysis, we included 24,893 women who were prescribed SSRIs/SNRIs and experienced an incident fracture. The median duration of exposure to SSRIs/SNRIs was 333 days. The results of the SCCS confirmed the primary results: Compared to baseline (non-exposure) periods, prescribing of SSRIs/SNRIs increased the risk of OFs during the first exposure window (day 1 to 30) by 38% (IRR = 1.38, 95% CI: 1.26–1.51) and by 58% in the second exposure window (day 31 to 90) (IRR = 1.58, 95% CI: 1.48–1.69). For the remaining exposure period, the risk was increased by 42% (IRR = 1.42, 95% CI: 1.37–1.48). Please see Table 4 for the results of the SCCS analysis.

**Table 4:** Results of SCCS analysis of periods of exposure to SSRIs/SNRIs compared with periods of no exposure

Risk window	Duration median (IQR)	Number of fractures	Crude IRR (95% CI)	Adjusted IRR (95% CI)*
Baseline period	634 (180–1708) days	10,664	Reference	Reference
Pre-exposure period	30 days	249	1.08 (0.95–1.23)	1.12 (0.98–1.27)
First exposure window (days 1 to 30)	30 days	499	1.29 (1.17–1.41)	1.38 (1.26–1.51)
Second exposure window (days 31 to 90)	60 days	1102	1.48 (1.39–1.58)	1.58 (1.48–1.69)
Remaining exposure period	333 (118–1098) days	10,045	1.43 (1.38–1.49)	1.42 (1.37–1.48)
Post exposure period	90 days	2334	1.33 (1.26–1.40)	1.26 (1.19–1.33)

*Adjusted for age

## Discussion

To the best of our knowledge, this is the first study to assess the risk of OFs in women prescribed SSRIs/SNRIs for common mental health problems. Menopausal women with CMHDs and prescribed SSRIs/SNRIs had a 32% increased risk of osteoporotic fractures compared to those not prescribed. Amongst women who were treated with SSRI/SNRI, those who were not prescribed MHT were at increased risk of OFs, whilst those on MHT were not at increased risk of OFs. However, we had few users of both MHT and SSRIs/SNRIs, and this subgroup analysis may have been underpowered to detect a difference. The risk of OFs peaked amongst aged below 50 years at 52% and slightly increased with increasing age in women aged 50 years and older. Longer duration of SSRIs/SNRIs prescribing was associated with a gradual increase in risk of OFs. Compared with women not prescribed SSRIs/SNRIs, the risk of OFs did not differ between prescribing SSRIs and SNRIs. The results of the secondary analysis in which a within-person study design was applied, confirmed those of the cohort study: The risk of OFs was increased in women prescribed SSRIs/SNRIs, and the risk increased during exposure to SSRIs/SNRIs compared with the baseline periods of no exposure. The risk increased by 38% during the first 30 days of exposure, 58% in days 31 to 90 of exposure, and 42% in the remaining exposure period to SSRIs/SNRIs.

This study contributes to literature reporting an increased risk of OFs in women with CMHDs prescribed SSRIs/SNRIs. This study showed a high risk of OFs in women of menopausal age prescribed SSRIs/SNRIs. Menopausal women are inherently at an increased risk of OFs, and symptoms of mental illness may increase this risk further [27, 28]. One plausible reason for the increased risk of fractures we reported in this study is that women prescribed SSRIs/SNRIs may have more severe mental health symptoms which may increase the associated risk of OFs [29]. However, the results of the self-controlled case series were not affected by confounding by indication as all women in this secondary analysis were exposed to SSRIs/SNRIs. The results of the secondary analysis confirmed the results of the cohort analysis. Moreover, the increased risk of OFs was sustained after introducing a 6-month lag period and excluding women with a history of osteoporosis. These observations suggest that the risk of OFs associated with SSRIs/SNRIs was a result of osteoporosis rather than the risk of falls associated with SSRIs/SNRIs. SSRIs/SNRIs may increase the risk of osteoporosis through their effect on osteoclast cells, responsible for decreasing bone density, via serotonin transporters [9, 10].

The risk of OFs was highest in women younger than 50 years old. Women younger than 50 years of age were included in the study if they had a record indicating menopause. Early menopausal women lose the protective effects of oestrogen for a longer period than women experiencing menopause after 50 years, which increases their vulnerability to OFs [30, 31]. To our knowledge, this is the first study to show the increased risk of OFs associated with prescribing of SSRIs/SNRIs in menopausal women younger than 50 years old.

When subgroup analysis was performed based on the duration of prescribing, the risk of OFs increased gradually with longer exposure to SSRIs/SNRIs. Women exposed to SSRIs/SNRIs for 5 years or longer had the highest risk of OFs. Ak et al. [32] investigated the association between duration of SSRIs use and bone mineral density in postmenopausal women. They used dual-energy X-ray absorptiometry at the lumbar spine and femoral neck. Their findings were compared to healthy volunteers. Consistent with our results, they found a negative correlation between the duration of SSRI use and bone mineral density [32]. This suggests that longer duration of SSRI use may be a risk factor for decreasing bone mineral density, potentially increasing the risk of OFs.

Despite the vulnerability of menopausal women to OFs [33], evidence on the risk of OFs associated with prescribing SSRIs/SNRIs remains scarce. Previous studies have focused on older adults without reporting the results in women or did not take any diagnoses of mental illness into consideration [8, 27, 28, 34]. Sheu et al. compared the use of SSRIs to the use of H2 antagonists or proton pump inhibitors in women without any mental illness. Similar to our results, rates of fractures were higher in women using SSRIs [12]. Nevertheless, results from this study cannot be applied to women with CMHDs as they were excluded from the analysis. Spangler et al. assessed the risk of OFs in menopausal women prescribed SSRIs and found a similar increase in the risk of fractures (HR = 1.30, 95% CI:1.20–1.55) [35]. However, this study included all SSRI users and relied on self-reported OF outcomes, which may have been affected by recall bias. Diem et al. assessed the risk of fractures in women greater than 65 years prescribed SSRIs. The inclusion age used was higher than the one used in our study. Similar to our results, SSRIs were associated with a 30% increase in the risk of non-spine fractures [36]. Bakken et al. found an increased risk of OFs associated with SSRIs, but contrary to our results, the excess risk of fractures decreased with increasing age. This study differs from our study in the inclusion of age and the outcome of fractures. The youngest inclusion age in their study was 60 years old, and they used hip fractures as the outcome [34]. Brannstrom et al. [26] examined the association between antidepressants and fractures in elderly women in the years before and after exposure to antidepressants using a study design resembling a cohort study. Contrary to our results, the highest risk of fractures was observed between days 16 and 30 before initiation of antidepressants (OR = 4.82, 95% CI: 3.85–6.02). The authors suggested that the risk of fractures in their study may have been the result of an indirect effect of uncontrolled depressive symptoms. However, this study differs from our study in both study design and population. Brannstrom et al. included all female antidepressant users over 65 years, whereas we included younger women with CMHDs after the menopausal transition. Our study population is likely to have been diagnosed with milder forms of mental illness than a general sample of all antidepressant users.

The risk of fractures associated with prescribing SSRIs/SNRIs was not examined previously in menopausal women using the SCCS design. Hubbard et al. examined the association between prescribing of SSRIs and the risk of hip fractures in the general population [37]. Similar to our results, the risk of OFs increased during the first 14 days of exposure to SSRIs (IR = 1.96) and remained high during the 90-day post-exposure period (IR = 1.57), after which the risk subsided. However, the authors did not assess the risk associated with prescribing of SNRIs. Coupland et al. assessed the risk of OFs associated with SSRIs and other antidepressants, including SNRIs, in individuals aged 65 years and over in a SCCS study [8]. However, in contrast to our results, the risk of OFs was highest in the initial 28 days of exposure and then slightly lowered in later periods of exposure, HR = 2.23 then HR = 1.38 for SSRIs and HR = 1.86 then HR = 1.12 for other antidepressants, respectively. Similar to our results, the risk of OFs remained increased in the first 84 days post-exposure to antidepressant treatment. Both studies by Coupland et al. and Hubbard et al. did not report the results for women separately, although baseline characteristics show that women make up the majority of their population [8, 37].

This is the first study to assess the risk of OFs associated with prescribing SSRIs/SNRIs to menopausal women using a large representative dataset from the UK population. This study had several limitations. Firstly, depressive symptoms that lead to a prescription for an SSRI/SNRI are associated with an increased risk of fractures [7]. Therefore, women using SSRIs/SNRIs may have confounding by indication. We minimised confounding by indication by comparing exposed and unexposed groups of menopausal women with CMHDs. Further, we used IPTW to balance measured characteristics between the groups. However, cohort studies cannot fully control for the potential residual risk of confounding by indication. In our study, women with CMHDs and prescribed SSRIs/SNRIs are likely to have had more severe disease than those not prescribed SSRIs/SNRIs. Our secondary analysis, in which we applied the SCCS, confirmed the results of the cohort study, and we therefore do not believe confounding by indication strongly affected our results. Secondly, the missing information in some covariates (smoking, body mass index, Townsend deprivation quintiles, and alcohol use) may have led to biassed estimates. Under the assumption that these variables were missing at random, we used multiple imputations to yield more specific effect estimates and minimise bias due to missing information. Thirdly, dispensing data were not recorded in the IMRD-UK database. Information on whether prescriptions were redeemed or consumed was unavailable. Thus, adherence to prescribed medications was not ensured. This was managed by considering women exposed to SSRIs/SNRIs if they had at least two consecutive prescriptions within a period of 180 days. Fourthly, factors that may affect the risk of OFs such as bone mineral density (BMD) and physical activity were not readily available in the IMRD database. Furthermore, ethnicity was planned to be included as a confounder because the risk of OFs differs between ethnic groups [38], but substantial missingness was found in ethnicity recordings that cannot be assumed to be missing at random. In addition, the use of calcium and vitamin D is indicative of osteoporosis leading to fractures and might not be completely recorded in the database, as they are available to be purchased over the counter. However, we included the diagnosis of osteoporosis, treatment of osteoporosis, and family history of osteoporosis as cofounders in our propensity score. Lastly, menopausal status, unless stated otherwise, was assumed based on age and was not confirmed. Patients with late menopause at 50–55 years old may have been misclassified and included as menopausal because we used the median age of menopause to define menopausal women. To control for this misclassification bias, sensitivity analyses were performed based on different assumed age of menopause onset, and the robustness of the study results was confirmed.

The additive risk of OFs in menopausal women prescribed SSRIs/SNRIs warrants the attention of both prescribers and women. Menopausal women with CMHDs are vulnerable to OFs, and SSRIs/SNRIs have an additive risk, particularly in women experiencing menopause before age 50. OFs in menopausal women are associated with a higher risk of mortality, and a burden of healthcare costs [39]. Prescribers need to assess menopausal women’s individual risk of OFs and discuss the risks to enable women to make informed decisions. Preventive measures include assessment of BMD, calcium and vitamin D supplementation [40], weight-bearing exercises, and prescribing of MHT [41]. Studies have shown that the use of menopausal hormone therapy (MHT) is beneficial in decreasing fracture risk in menopausal women [42–44]. Use of MHT during perimenopause on its own or as add-on therapy to antidepressants has been suggested as an option to improve treatment efficacy [45]. However, it is unknown if MHT mitigates the risk of fractures if combined with SSRIs/SNRIs. The current study did not have enough power to adequately investigate the interaction between the risk of fractures and prescribing of SSRIs/SNRIs antidepressants in combination with MHT to menopausal women with CMHDs. We did not find an increased risk of OFs in women prescribed MHT in addition to SSRIs/SNRIs, but further investigative studies need to be done to verify the ability of MHT to mitigate the risk of OFs in menopausal women with CMHDs and prescribed SSRIs/SNRIs. For women in whom the prescribing of MHT is indicated, treatment has the additional benefit of potentiating the antidepressant response to SSRIs/SNRIs and controlling vasomotor symptoms which subsequently may improve mental health symptoms [45, 46]. For most women who start MHT within 10 years of menopause benefits outweigh risks [41]. Finally, our study showed that longer durations of treatment with SSRIs/SNRIs are associated with an increased risk of OFs; prescribers need to consider the benefit-risk ratio for prescribing SSRIs/SNRIs for prolonged periods. If needed, prescribers should consider screening for osteoporosis in women using SSRIs/SNRIs for more than 5 years.

## Conclusion

Prescribing SSRIs/SNRIs to menopausal women with common mental health diagnoses appears to be associated with an increased risk of osteoporotic fractures; the associated risk is more pronounced in women < 50 years and in women prescribed SSRIs/SNRIs for longer than 5 years. Menopausal women with common mental health diagnoses and prescribed SSRIs/SNRIs should be evaluated for their risk of osteoporosis, and preventive measures should be considered to mitigate the risk of osteoporotic fractures.

## Data Availability

The current study was approved by the IQVIA Medical Research Data Scientific Review Committee on 14 March 2022 (Reference number: 22SRC006_A2).

## Appendix

Table 5

**Table 5:** Code list for study medications

Drug code	Medication
91395998	Citalopram
93996990	
95667990	
95334990	
91380998	
94895990	
95271990	
95995979	
95269990	
93994990	
94937990	
93946990	
95420990	
95979979	
94880990	
95418990	
95631990	
95984979	
87251998	
95333990	
95633990	
95705990	
95666990	
93948990	
92174998	
91395996	
94894990	
94603990	
94936990	
93947990	
95335990	
95632990	
95704990	
95703990	
94893990	
95668990	
91380997	
95270990	
95421990	
91395997	
91380996	
92172998	
86996998	Duloxetine
37600978	
86998998	
89023979	
51109978	
86999998	
86997998	
39667978	
88285998	Escitalopram
82790998	
82791998	
87662998	
87663998	
89381979	
85970998	
89383979	
98561998	
91671998	
85971998	
98088998	
84436998	Fluoxetine
96155979	
96606990	
94447998	
94447997	
94447996	
99592998	
96729990	
90766998	
91923990	
93066990	
96659990	
96643990	
96651990	
96654990	
90814998	
94490997	
96674990	
96647990	
76398978	
96162979	
96143979	
75904978	
95813990	
93905990	
96168979	
96161979	
96709990	
91928990	
95426990	
84403998	
94490998	
96281990	
95388990	
96644990	
96272990	
90159998	
94490996	
75905978	
95610990	
95820990	
96493998	Fluvoxamine
96810989	
96492998	
96093990	
96493997	
96492997	
96345989	
96068979	Paroxetine
93490998	
95028990	
93490996	
93490997	
96082979	
54495979	
95051990	
84807998	
95332990	
95007990	
93489998	
95350990	
93489997	
96087990	
96070979	
54494979	
93489996	
66541979	
95578990	
93487990	
85382998	
94852990	
96098979	
88838998	
93749990	Sertraline
93174998	
92729990	
93173997	
96114979	
93843990	
93694990	
86159998	
93174997	
60187979	
60188979	
52706979	
92728990	
93733990	
93173998	
93732990	
93752990	
93753990	
96118979	
93842990	
96136979	
81930998	Venlafaxine
80024978	
83217998	
96059979	
92597990	
96029979	
88776997	
83145998	
82959998	
83149998	
83205998	
39137978	
83218998	
83204998	
83264998	
96024979	
83209998	
98336996	
81506998	
83160998	
86431998	
81505998	
83146998	
82963998	
82962998	
96023979	
82190998	
99896996	
83115998	
83265998	
98336998	
96054979	
88776998	
83159998	
83075998	
98336997	
83163998	
88755997	
96052979	
92596990	
96022979	
81749998	
81750998	
99896997	
82540998	
96041979	
96065979	
39138978	
83114998	
82874998	
83150998	
83162998	
82191998	
96036979	
83210998	
96034979	
81929998	
88755998	
83157998	
82961998	
52700979	
82875998	
83158998	
52164979	
80023978	
83074998	
96033979	
52165979	
99896998	

Table 6

**Table 6:** Code list for osteoporotic fractures

Medical code	Description	Type
S34..00	Fracture of ankle	Ankle
S34x.00	Closed fracture ankle, unspecified	Ankle
S348.00	Fracture of medial malleolus	Ankle
7K1L800	Closed reduction of fracture of ankle	Ankle
S340.00	Closed fracture ankle, medial malleolus	Ankle
S349.00	Fracture of lateral malleolus	Ankle
S342000	Closed fracture ankle, lateral malleolus, low	Ankle
S344.00	Closed fracture ankle, bimalleolar	Ankle
S342.00	Closed fracture ankle, lateral malleolus	Ankle
S34z.00	Fracture of ankle, NOS	Ankle
S346.00	Closed fracture ankle, trimalleolar	Ankle
S344.11	Dupuytren’s fracture, fibula	Ankle
S4G..00	Fracture-dislocation or subluxation ankle	Ankle
S4G0.00	Closed fracture-dislocation, ankle joint	Ankle
S342100	Closed fracture ankle, lateral malleolus, high	Ankle
S344000	Closed fracture ankle, bimalleolar, low fibular fracture	Ankle
S346100	Closed fracture ankle, trimalleolar, high fibular fracture	Ankle
S344100	Closed fracture ankle, bimalleolar, high fibular fracture	Ankle
S346000	Closed fracture ankle, trimalleolar, low fibular fracture	Ankle
S4G2.00	Closed fracture-subluxation, ankle joint	Ankle
S347.00	Open fracture ankle, trimalleolar	Ankle open
S343.00	Open fracture ankle, lateral malleolus	Ankle open
S345.00	Open fracture ankle, bimalleolar	Ankle open
S4G3.00	Open fracture-subluxation, ankle joint	Ankle open
S341.00	Open fracture ankle, medial malleolus	Ankle open
S34y.00	Open fracture ankle, unspecified	Ankle open
S343000	Open fracture ankle, lateral malleolus, low	Ankle open
S347000	Open fracture ankle, trimalleolar, low fibular fracture	Ankle open
S4G1.00	Open fracture-dislocation, ankle joint	Ankle open
S345000	Open fracture ankle, bimalleolar, low fibular fracture	Ankle open
S343100	Open fracture ankle, lateral malleolus, high	Ankle open
S345100	Open fracture ankle, bimalleolar, high fibular fracture	Ankle open
S347100	Open fracture ankle, trimalleolar, high fibular fracture	Ankle open
S224.11	Elbow fracture—closed	Elbow
S228.00	Fracture of lower end of humerus	Elbow
S237.00	Fracture of upper end of radius	Elbow
S224100	Closed fracture distal humerus, supracondylar	Elbow
S230600	Closed fracture radius, head	Elbow
7K1LE00	Closed reduction of fracture of elbow	Elbow
S224600	Closed fracture distal humerus, lateral epicondyle	Elbow
S4B0000	Closed fracture-dislocation elbow joint	Elbow
S230100	Closed fracture olecranon, extra-articular	Elbow
S224.00	Closed fracture of the distal humerus	Elbow
S224200	Closed fracture distal humerus, lateral condyle	Elbow
S224800	Closed fracture distal humerus, capitellum	Elbow
S224700	Closed fracture distal humerus, medial epicondyle	Elbow
S224400	Closed fracture of distal humerus, condyle(s) unspecified	Elbow
S236.00	Fracture of upper end of ulna	Elbow
S224z00	Closed fracture of distal humerus, not otherwise specified	Elbow
S4A0000	Closed fracture-dislocation shoulder joint	Elbow
S224000	Closed fracture of elbow, unspecified part	Elbow
S224300	Closed fracture distal humerus, medial condyle	Elbow
S224500	Closed fracture of distal humerus, trochlea	Elbow
S224900	Closed fracture distal humerus, bicondylar (T-Y fracture)	Elbow
S4B2.00	Closed fracture-subluxation elbow	Elbow
S224 × 00	Closed fracture of distal humerus, multiple	Elbow
S4B2000	Closed fracture-subluxation elbow joint	Elbow
S225.11	Elbow fracture—open	Elbow open
S4B3.00	Open fracture-subluxation elbow	Elbow open
S312300	Closed fracture distal femur, supracondylar	Femur distal
S312200	Closed fracture of femur, lower epiphysis	Femur distal
S312.11	Closed fracture of femur, distal end	Femur distal
S312.00	Closed fracture distal femur	Femur distal
S4F4.00	Closed fracture-dislocation, patello-femoral joint	Femur distal
S312500	Closed fracture distal femur, lateral condyle	Femur distal
S4F6.00	Closed fracture-subluxation, patello-femoral joint	Femur distal
S312400	Closed fracture distal femur, medial condyle	Femur distal
S312000	Closed fracture of distal femur, unspecified	Femur distal
S312600	Closed fracture distal femur, bicondylar (T-Y fracture)	Femur distal
S312 × 00	Closed fracture distal femur, comminuted/intra-articular	Femur distal
S312z00	Closed fracture of distal femur not otherwise specified	Femur distal
S35..11	Metatarsal bone fracture	Foot
S35..00	Fracture of one or more tarsal and metatarsal bones	Foot
S352.11	March fracture	Foot
S36..11	Toe fracture	Foot
S354.00	Fracture of calcaneus	Foot
S362.00	Fracture of great toe	Foot
S355.00	Fracture of talus	Foot
S36..00	Fracture of one or more phalanges of foot	Foot
S35..12	Tarsal bone fracture	Foot
S352700	Closed fracture metatarsal	Foot
S360.00	Closed fracture of one or more phalanges of foot	Foot
S352300	Closed fracture cuboid	Foot
S356.00	Fracture of metatarsal bone	Foot
S363.00	Fracture of other toe	Foot
S350.00	Closed fracture of calcaneus	Foot
S350.11	Heel bone fracture	Foot
S3 × 4.00	Multiple fractures of foot	Foot
S352200	Closed fracture navicular	Foot
S352100	Closed fracture of talus	Foot
S352.00	Closed fracture of other tarsal and metatarsal bones	Foot
S4H..00	Fracture-dislocation or subluxation foot	Foot
S4H2300	Closed #-subluxation, metatarsophalangeal joint, single	Foot
S352000	Closed fracture of tarsal bone, unspecified	Foot
S352B00	Closed fracture metatarsal base	Foot
S360000	Closed fracture proximal phalanx, toe	Foot
S352C00	Closed fracture metatarsal shaft	Foot
S360200	Closed fracture distal phalanx, toe	Foot
S352F00	Closed fracture metatarsal, multiple	Foot
S362000	Closed fracture of great toe	Foot
S4H0400	Closed fracture-dislocation, IPJ, single toe	Foot
S352J00	Closed fracture of base of fifth metatarsal	Foot
S352D00	Closed fracture metatarsal neck	Foot
S360100	Closed fracture middle phalanx, toe	Foot
S36z.00	Fracture of one or more phalanges of foot NOS	Foot
S350100	Closed fracture calcaneus, intra-articular	Foot
S352400	Closed fracture medial cuneiform	Foot
S352E00	Closed fracture metatarsal head	Foot
S352H00	Closed fracture of cuneiforms	Foot
S352800	Closed fracture talus, head	Foot
S35z.00	Fracture of tarsal and metatarsal bones NOS	Foot
S4H0300	Closed #-dislocation, metatarsophalangeal joint, single	Foot
S352z00	Closed fracture of one or more tarsal + metatarsal bones NOS	Foot
S4H0.00	Closed fracture-dislocation foot	Foot
S4H2.00	Closed fracture-subluxation, foot	Foot
S352A00	Closed fracture talus, body	Foot
S352900	Closed fracture talus, neck	Foot
S352600	Closed fracture lateral cuneiform	Foot
S4H0500	Closed #-dislocation, metatarsophalangeal joint, multiple	Foot
S360300	Closed fracture multiple phalanges, toe	Foot
S4H0000	Closed fracture-dislocation, subtalar joint	Foot
S4H2500	Closed #-subluxation, metatarsophalangeal joint, multiple	Foot
S350000	Closed fracture calcaneus, extra-articular	Foot
S352500	Closed fracture intermediate cuneiform	Foot
S352G00	Closed tarsal fractures, multiple	Foot
S4H2100	Closed fracture-subluxation, midtarsal joint	Foot
S4H0200	Closed fracture-dislocation, tarsometatarsal joint	Foot
S4H0100	Closed fracture-dislocation, midtarsal joint	Foot
S4H2000	Closed fracture-subluxation, subtalar joint	Foot
S4H2600	Closed fracture-subluxation, IPJ, multiple toes	Foot
S4H2200	Closed fracture-subluxation, tarsometatarsal joint	Foot
S4H0600	Closed fracture-dislocation, IPJ, multiple toes	Foot
S4H2400	Closed fracture-subluxation, IPJ, single toe	Foot
Syu9400	[X]Fracture of other tarsal bones	Foot
S362100	Open fracture of great toe	Foot open
S361.00	Open fracture of one or more phalanges of foot	Foot open
S353100	Open fracture of talus	Foot open
S4H1300	Open fracture-dislocation, metatarsophalangeal joint, single	Foot open
S4H1000	Open fracture-dislocation, subtalar joint	Foot open
S353200	Open fracture navicular	Foot open
S4H1400	Open fracture-dislocation, IPJ, single toe	Foot open
S351.00	Open fracture of calcaneus	Foot open
S353z00	Open fracture of tarsal and metatarsal bones NOS	Foot open
S353300	Open fracture cuboid	Foot open
S361000	Open fracture proximal phalanx, toe	Foot open
S361200	Open fracture distal phalanx, toe	Foot open
S4H1.00	Open fracture-dislocation, foot	Foot open
S353700	Open fracture metatarsal	Foot open
S361300	Open fracture multiple phalanges, toe	Foot open
S353C00	Open fracture metatarsal shaft	Foot open
S4H1500	Open #-dislocation, metatarsophalangeal joint, multiple	Foot open
S4H3300	Open fracture-subluxation, metatarsophalangeal joint, single	Foot open
S353F00	Open fracture metatarsal, multiple	Foot open
S353400	Open fracture medial cuneiform	Foot open
S353.00	Open fracture of other tarsal and metatarsal bones	Foot open
S353J00	Open fracture of base of fifth metatarsal	Foot open
S4H3400	Open fracture-subluxation, IPJ, single toe	Foot open
S361100	Open fracture middle phalanx, toe	Foot open
S351100	Open fractures calcaneus, intra-articular	Foot open
S353B00	Open fracture metatarsal base	Foot open
S4H1200	Open fracture-dislocation, tarsometatarsal joint	Foot open
S353900	Open fracture talus, neck	Foot open
S4H3.00	Open fracture-subluxation, foot	Foot open
S353500	Open fracture intermediate cuneiform	Foot open
S353A00	Open fracture talus, body	Foot open
S353D00	Open fracture metatarsal neck	Foot open
S353000	Open fracture of tarsal bone, unspecified	Foot open
S353E00	Open fracture metatarsal head	Foot open
S32..00	Fracture of patella	Knee
7K1L600	Closed reduction of fracture of knee	Knee
S320.00	Closed fracture of the patella	Knee
S4F2.00	Closed fracture-subluxation, knee joint	Knee
S32z.00	Fracture of patella, NOS	Knee
S4F0.00	Closed fracture-dislocation, knee joint	Knee
S320400	Closed fracture patella, comminuted (stellate)	Knee
S320200	Closed fracture patella, distal pole	Knee
S320000	Closed fracture patella, transverse	Knee
S320100	Closed fracture patella, proximal pole	Knee
S320300	Closed fracture patella, vertical	Knee
S10B600	Multiple fractures of lumbar spine and pelvis	Lumbar spine pelvis
S10B.00	Fracture of lumbar spine and pelvis	Lumbar spine pelvis
S20..00	Fracture of clavicle	Non hip other
S22..00	Fracture of humerus	Non hip other
S31z.00	Fracture of femur, NOS	Non hip other
S339.00	Fracture of fibula alone	Non hip other
S21..00	Fracture of scapula	Non hip other
S28..11	Ill-defined fracture of arm	Non hip other
S3…11	Leg fracture	Non hip other
S28z.00	Ill-defined fractures of upper limb NOS	Non hip other
S122.00	Closed fracture sternum	Non hip other
S210300	Closed fracture scapula, glenoid	Non hip other
S20..11	Collar bone fracture	Non hip other
S33 × 100	Closed fracture of fibula, unspecified part, NOS	Non hip other
S242300	Multiple fractures of metacarpal bones	Non hip other
S210400	Closed fracture scapula, blade	Non hip other
S2…11	Arm fracture	Non hip other
S2…00	Fracture of upper limb	Non hip other
7K1J.00	Closed (or no) reduction of fracture and internal fixation	Non hip other
S312100	Closed fracture of femoral condyle, unspecified	Non hip other
S339000	Closed fracture of distal fibula	Non hip other
S310.00	Closed fracture of femur, shaft or unspecified part	Non hip other
S31..00	Other fracture of femur	Non hip other
S315.00	Fracture of lower end of femur	Non hip other
ZV67400	[V]Fracture follow-up	Non hip other
7K1LJ00	Closed reduction of fracture of thumb	Non hip other
S3…00	Fracture of lower limb	Non hip other
S3 × 3.00	Multiple fractures of lower leg	Non hip other
S22z.00	Fracture of humerus NOS	Non hip other
S21..11	Shoulder blade fracture	Non hip other
S3X..00	Fracture of lower leg, part unspecified	Non hip other
7K1LB00	Closed reduction of fracture of hallux	Non hip other
N331M00	Fragility fracture due to unspecified osteoporosis	Non hip other
S128.00	Fracture of sternum	Non hip other
S230B00	Closed fracture olecranon, intra-articular	Non hip other
S210100	Closed fracture scapula, acromion	Non hip other
S292.00	Multiple fractures of clavicle, scapula and humerus	Non hip other
NyuB800	[X]Unspecified osteoporosis with pathological fracture	Non hip other
7K1L500	Closed reduction of fracture of femur	Non hip other
S222000	Closed fracture of humerus NOS	Non hip other
S310012	Upper leg fracture NOS	Non hip other
S4A2100	Closed fracture-subluxation acromio-clavicular joint	Non hip other
7K1K.00	Closed (or no) reduction of fracture and external fixation	Non hip other
N331600	Idiopathic osteoporosis with pathological fracture	Non hip other
S210.00	Closed fracture of scapula	Non hip other
S4A0100	Closed fracture-dislocation acromio-clavicular joint	Non hip other
S12z.12	Sternum fracture NOS	Non hip other
S20z.00	Fracture of clavicle NOS	Non hip other
S200z00	Closed fracture of clavicle NOS	Non hip other
S200200	Closed fracture clavicle, shaft	Non hip other
S332100	Closed fracture shaft of fibula	Non hip other
S200300	Closed fracture clavicle, lateral end	Non hip other
S2z..00	Fracture of upper limb NOS	Non hip other
S352111	Closed fracture of astragalus	Non hip other
S227.00	Fracture of shaft of humerus	Non hip other
S29..11	Multiple fractures of arm	Non hip other
N331300	Osteoporosis of disuse with pathological fracture	Non hip other
S330100	Closed fracture proximal fibula	Non hip other
S200.00	Closed fracture of clavicle	Non hip other
S21z.00	Fracture of scapula NOS	Non hip other
S2A..00	Fracture of upper limb, level unspecified	Non hip other
S37..00	Fracture of lower limb, level unspecified	Non hip other
S210200	Closed fracture scapula, coracoid	Non hip other
S33x.11	Lower leg fracture NOS	Non hip other
S210600	Closed fracture scapula, neck	Non hip other
S310000	Closed fracture of femur, unspecified part	Non hip other
S210000	Closed fracture of scapula, unspecified part	Non hip other
7K1LN00	Closed reduction of fracture of upper limb	Non hip other
N331B00	Postmenopausal osteoporosis with pathological fracture	Non hip other
S370.00	Closed fracture of lower limb, level unspecified	Non hip other
S292000	Closed multiple fractures of clavicle, scapula and humerus	Non hip other
S232100	Closed fracture of the radial shaft	Non hip other
S200100	Closed fracture clavicle, medial end	Non hip other
S200000	Closed fracture of clavicle, unspecified part	Non hip other
N331500	Drug-induced osteoporosis with pathological fracture	Non hip other
S280.00	Closed ill-defined fractures of upper limb	Non hip other
ZV66400	[V]Convalescence after treatment of fracture	Non hip other
S210500	Closed fracture scapula, spine	Non hip other
S28..00	Ill-defined fractures of upper limb	Non hip other
S310z00	Closed fracture of shaft or unspecified part, NOS	Non hip other
S330900	Closed fracture fibula, neck	Non hip other
S330800	Closed fracture fibula, head	Non hip other
Zw01.00	[Q] Fractures involving the epiphyseal plate	Non hip other
S12X.00	Fracture of bony thorax, part unspecified	Non hip other
7K1LC00	Closed reduction of fracture of lower limb	Non hip other
SR16000	Closed fracture inv thorax wth low back and pelvis and limbs	Non hip other
NyuB000	[X]Other osteoporosis with pathological fracture	Non hip other
S210z00	Closed fracture of scapula NOS	Non hip other
0	Closed fracture of bony thorax part unspecified	Non hip other
S4J2000	Closed fracture-subluxation of sternum	Non hip other
S4J0000	Closed fracture-dislocation of sternum	Non hip other
S29..13	Multiple fractures of sternum	Non hip other
Syu7200	[X]Fractures of other parts of femur	Non hip other
SR1z000	[X]Closed multiple fractures unspecified	Non hip other
SR12000	Closed fractures involving multiple regions of one upp limb	Non hip other
N331N00	Fragility fracture	Non hip other
N331M11	Minimal trauma fracture due to unspecified osteoporosis	Non hip other
N331N11	Minimal trauma fracture	Non hip other
Zw02500	[Q] Refracture	Non hip other
S294000	Cl fractures involving multiple regions of both upper limbs	Non hip other
S12y000	Closed fracture of other parts of bony thorax	Non hip other
Syu8300	[X]Fractures of other parts of lower leg	Non hip other
Syu4200	[X]Multiple fractures of clavicle, scapula and humerus	Non hip other
S12y.00	Fracture of other parts of bony thorax	Non hip other
Syu8D00	[X]Fracture of lower leg, part unspecified	Non hip other
SR15000	Cl fractures involving multiple regions upper with lower lmb	Non hip other
SR1z100	[X]Open multiple fractures unspecified	Non hip other open
S106000	Closed compression fracture sacrum	Pelvis
S130.00	Closed fracture acetabulum	Pelvis
S130z00	Closed fracture acetabulum NOS	Pelvis
S130200	Closed fracture acetabulum, anterior column	Pelvis
S130000	Closed fracture acetabulum, anterior lip alone	Pelvis
S130600	Closed fracture acetabulum, double column unspecified	Pelvis
S130400	Closed fracture acetabulum, floor	Pelvis
S130300	Closed fracture acetabulum, posterior column	Pelvis
S130100	Closed fracture acetabulum, posterior lip alone	Pelvis
S134800	Closed fracture dislocation of sacro-iliac joint	Pelvis
S134000	Closed fracture of ilium, unspecified	Pelvis
S13y.00	Closed fracture of pelvis NOS	Pelvis
S134500	Closed fracture pelvis, anterior inferior iliac spine	Pelvis
S134400	Closed fracture pelvis, anterior superior iliac spine	Pelvis
S108.00	Closed fracture pelvis, coccyx	Pelvis
S134600	Closed fracture pelvis, iliac wing	Pelvis
S134300	Closed fracture pelvis, ischial tuberosity	Pelvis
S134100	Closed fracture pelvis, ischium	Pelvis
S132100	Closed fracture pelvis, multiple pubic rami—stable	Pelvis
S132200	Closed fracture pelvis, multiple pubic rami—unstable	Pelvis
S132000	Closed fracture pelvis, single pubic ramus	Pelvis
S132.00	Closed fracture pubis	Pelvis
S132z00	Closed fracture pubis NOS	Pelvis
S106.00	Closed fracture sacrum	Pelvis
S4J0100	Closed fracture-dislocation of pelvis	Pelvis
S4J2100	Closed fracture-subluxation of pelvis	Pelvis
S134700	Closed vertical fracture of ilium	Pelvis
S106100	Closed vertical fracture of sacrum	Pelvis
S10B400	Fracture of acetabulum	Pelvis
S10B200	Fracture of coccyx	Pelvis
S10B300	Fracture of ilium	Pelvis
S10B500	Fracture of pubis	Pelvis
S10B100	Fracture of sacrum	Pelvis
S13..00	Fracture or disruption of pelvis	Pelvis
S134.00	Other or multiple closed fracture of pelvis	Pelvis
S134z00	Other or multiple closed fracture of pelvis NOS	Pelvis
S130y00	Other specified closed fracture acetabulum	Pelvis
S132y00	Other specified closed fracture pubis	Pelvis
S13z.00	Open fracture of pelvis NOS	Pelvis open
S107.00	Open fracture sacrum	Pelvis open
S118.00	Closed fracture of coccyx with spinal cord lesion	Pelvis spinal cord
S116.00	Closed fracture of sacrum with spinal cord lesion	Pelvis spinal cord
S116z00	Closed fracture of sacrum with spinal cord lesion NOS	Pelvis spinal cord
S118z00	Closed fracture of coccyx with spinal cord lesion NOS	Pelvis spinal cord
S120900	Closed fracture multiple ribs	Rib
S120800	Closed fracture of eight or more ribs	Rib
S120500	Closed fracture of five ribs	Rib
S120400	Closed fracture of four ribs	Rib
S120100	Closed fracture of one rib	Rib
S120z00	Closed fracture of rib(s) NOS	Rib
S120000	Closed fracture of rib, unspecified	Rib
S120700	Closed fracture of seven ribs	Rib
S120600	Closed fracture of six ribs	Rib
S120300	Closed fracture of three ribs	Rib
S120200	Closed fracture of two ribs	Rib
S120.00	Closed fracture rib	Rib
S127100	Cough fracture of ribs	Rib
S127000	Multiple fractures of ribs	Rib
S29..12	Multiple rib fractures	Rib
S12z.11	Rib fracture NOS	Rib
S127.00	Fracture of rib	Rib
S120A00	Cough fracture	Rib
S12..00	Fracture of rib(s), sternum, larynx and trachea	Rib
S12z.00	Fracture of rib(s), sternum, larynx or trachea NOS	Rib
S226.00	Fracture of upper end of humerus	Shoulder
7K1LF00	Closed reduction of fracture of humerus	Shoulder
7K1LG00	Closed reduction of fracture of shoulder	Shoulder
S4A0.00	Closed fracture-dislocation shoulder	Shoulder
S220300	Closed fracture proximal humerus, greater tuberosity	Shoulder
S220.00	Closed fracture of the proximal humerus	Shoulder
S220100	Closed fracture proximal humerus, neck	Shoulder
S222100	Closed fracture of humerus, shaft	Shoulder
S220400	Closed fracture proximal humerus, head	Shoulder
S220700	Closed fracture proximal humerus, four part	Shoulder
S220200	Closed fracture of proximal humerus, anatomical neck	Shoulder
S4A2.00	Closed fracture-subluxation shoulder	Shoulder
S222.00	Closed fracture of humerus, shaft or unspecified part	Shoulder
S220z00	Closed fracture of proximal humerus not otherwise specified	Shoulder
S220600	Closed fracture proximal humerus, three part	Shoulder
S220000	Closed fracture of proximal humerus, unspecified part	Shoulder
S220500	Closed fracture of humerus, upper epiphysis	Shoulder
Syu4300	[X]Fracture of other parts of shoulder and upper arm	Shoulder
Syu4400	[X]Fracture of shoulder and upper arm, unspecified	Shoulder
S4A2000	Closed fracture-subluxation shoulder joint	Shoulder
S222z00	Closed fracture of humerus, shaft or unspecified part NOS	Shoulder
S221.11	Shoulder fracture—open	Shoulder open
S221000	Open fracture of proximal humerus, unspecified part	Shoulder open
S104.00	Closed fracture lumbar vertebra	Spine
S104000	Closed fracture lumbar vertebra, burst	Spine
S104500	Closed fracture lumbar vertebra, posterior arch	Spine
S104300	Closed fracture lumbar vertebra, spinous process	Spine
S104200	Closed fracture lumbar vertebra, spondylolysis	Spine
S104400	Closed fracture lumbar vertebra, transverse process	Spine
S104600	Closed fracture lumbar vertebra, tricolumnar	Spine
S104100	Closed fracture lumbar vertebra, wedge	Spine
S114.00	Closed fracture of lumbar spine with spinal cord lesion	Spine
S11x.00	Closed fracture of spine with spinal cord lesion unspecified	Spine
S10x.00	Closed fracture of spine, unspecified,	Spine
S112z00	Closed fracture of thoracic spine with cord lesion NOS	Spine
S112.00	Closed fracture of thoracic spine with spinal cord lesion	Spine
S102.00	Closed fracture thoracic vertebra	Spine
S102z00	Closed fracture thoracic vertebra not otherwise specified	Spine
S102000	Closed fracture thoracic vertebra, burst	Spine
S102500	Closed fracture thoracic vertebra, posterior arch	Spine
S102300	Closed fracture thoracic vertebra, spinous process	Spine
S102200	Closed fracture thoracic vertebra, spondylolysis	Spine
S102400	Closed fracture thoracic vertebra, transverse process	Spine
S102100	Closed fracture thoracic vertebra, wedge	Spine
S150000	Closed multiple fractures of thoracic spine	Spine
S114500	Closed spinal fracture with cauda equina lesion	Spine
S114100	Closed spinal fracture with complete lumbar cord lesion	Spine
S114000	Closed spinal fracture with unspecified lumbar cord lesion	Spine
S112700	Cls spinal fracture with complete thorac cord lesion, T7-12	Spine
S112A00	Cls spinal fracture with posterior thorac cord lesion, T7-12	Spine
S112600	Cls spinal fracture with unspec thoracic cord lesion, T7-12	Spine
S112100	Cls spinal fracture wth complete thoracic cord lesion, T1-6	Spine
N1y1.00	Fatigue fracture of vertebra	Spine
S150.00	Multiple fractures of thoracic spine	Spine
N331A00	Osteoporosis + pathological fracture cervical vertebrae	Spine
N331800	Osteoporosis + pathological fracture lumbar vertebrae	Spine
N331900	Osteoporosis + pathological fracture thoracic vertebrae	Spine
7J41500	Balloon kyphoplasty of fracture of spine	Spine
S112000	Cls spinal fracture with unspec thoracic cord lesion, T1-6	Spine
S10B000	Fracture of lumbar vertebra	Spine
S10..00	Fracture of spine without mention of spinal cord injury	Spine
S10z.00	Fracture of spine without mention of spinal cord lesion NOS	Spine
S15..00	Fracture of thoracic vertebra	Spine
S10..12	Fracture of vertebra without spinal cord lesion	Spine
S102y00	Other specified closed fracture thoracic vertebra	Spine
N331100	Pathological fracture of lumbar vertebra	Spine
N331000	Pathological fracture of thoracic vertebra	Spine
7J42600	Primary bedrest stabilisation of spinal fracture	Spine
7J42900	Primary cast stabilisation of spinal fracture	Spine
7J41300	Vertebroplasty of fracture of spine	Spine
S113.00	Open fracture of thoracic spine with spinal cord lesion	Spine open
S10y.00	Open fracture of spine, unspecified,	Spine open
S150100	Open multiple fracture of thoracic spine	Spine open
S33 × 000	Closed fracture of tibia, unspecified part, NOS	Tibia
S33..00	Fracture of tibia and fibula	Tibia
S33 × 200	Closed fracture of tibia and fibula, unspecified part	Tibia
7K1L700	Closed reduction of fracture of tibia and or fibula	Tibia
S337.00	Fracture of shaft of tibia	Tibia
S334100	Closed fracture distal tibia, intra-articular	Tibia
S338.00	Fracture of lower end of tibia	Tibia
S33z.00	Fracture of tibia and fibula, NOS	Tibia
S334.00	Closed fracture distal tibia	Tibia
S33x.00	Closed fracture of tibia and fibula, unspecified part, NOS	Tibia
S332.00	Closed fracture of tibia/fibula, shaft	Tibia
S332200	Closed fracture of tibia and fibula, shaft	Tibia
S332000	Closed fracture shaft of tibia	Tibia
S334000	Closed fracture distal tibia, extra-articular	Tibia
S33xz00	Closed fracture of tibia and fibula, unspecified part, NOS	Tibia
S332z00	Closed fracture of tibia and fibula, shaft, NOS	Tibia
S33A.00	Fracture of tibia	Tibia
S33C.00	Closed fracture of distal tibia and fibula	Tibia
Q203300	Fracture of tibia or fibula due to birth trauma	Tibia birth
S333200	Open fracture of tibia and fibula, shaft	Tibia open
S335000	Open fracture distal tibia, extra-articular	Tibia open
S335.00	Open fracture distal tibia	Tibia open
S333.00	Open fracture of tibia/fibula, shaft	Tibia open
S333000	Open fracture shaft of tibia	Tibia open
S333z00	Open fracture of tibia and fibula, shaft, NOS	Tibia open
S33y.00	Open fracture of tibia and fibula, unspecified part, NOS	Tibia open
S33y200	Open fracture of tibia and fibula, unspecified part	Tibia open
S33y000	Open fracture of tibia, unspecified part, NOS	Tibia open
S331000	Open fracture of the proximal tibia	Tibia open
S331.00	Open fracture of tibia and fibula, proximal	Tibia open
S331300	Open fracture proximal tibia, medial condyle (plateau)	Tibia open
S331400	Open fracture proximal tibia, lateral condyle (plateau)	Tibia open
S331700	Open fracture tubercle, tibia	Tibia open
S331012	Open fracture of tibial tuberosity	Tibia open
S331200	Open fracture of tibia and fibula, proximal	Tibia open
S331z00	Open fracture of tibia and fibula, proximal NOS	Tibia open
S33yz00	Open fracture of tibia and fibula, unspecified part, NOS	Tibia open
S331600	Open fracture spine, tibia	Tibia open
S335100	Open fracture distal tibia, intra-articular	Tibia open
S331011	Open fracture of tibial condyles	Tibia open
S33B.00	Open fracture of distal tibia and fibula	Tibia open
S331A00	Open fracture tibial plateau	Tibia open
S336.00	Fracture of upper end of tibia	Tibia prox
S330300	Closed fracture proximal tibia, medial condyle (plateau)	Tibia prox
S330400	Closed fracture proximal tibia, lateral condyle (plateau)	Tibia prox
S330012	Closed fracture of tibial tuberosity	Tibia prox
S330000	Closed fracture of the proximal tibia	Tibia prox
S330600	Closed fracture spine, tibia	Tibia prox
S330700	Closed fracture tubercle, tibia	Tibia prox
S330500	Closed fracture proximal tibia, bicondylar	Tibia prox
S330z00	Closed fracture of tibia and fibula, proximal NOS	Tibia prox
S330.00	Closed fracture of tibia and fibula, proximal	Tibia prox
S330011	Closed fracture of tibial condyles	Tibia prox
S330200	Closed fracture of tibia and fibula, proximal	Tibia prox
S336000	Fracture tibial plateau	Tibia prox
S233.00	Open fracture of radius and ulna, shaft	Radius ulna open
S233z00	Open fracture of radius and ulna, shaft, NOS	Radius ulna open
S233300	Open fracture radius and ulna, middle	Radius ulna open
S233000	Open fracture of radius, shaft, unspecified	Radius ulna open
S233100	Open fracture of the radial shaft	Radius ulna open
S23 × 111	Fracture of radius NOS	Wrist forearm
S23B.00	Fracture of lower end of radius	Wrist forearm
S234.11	Wrist fracture—closed	Wrist forearm
S234100	Closed Colles’ fracture	Wrist forearm
S23z.00	Fracture of radius and ulna, NOS	Wrist forearm
S23 × 211	Fracture of ulna NOS	Wrist forearm
S234200	Closed fracture of the distal radius, unspecified	Wrist forearm
S230300	Closed Monteggia’s fracture	Wrist forearm
S234700	Closed Smith’s fracture	Wrist forearm
S23 × 300	Closed fracture of the radius and ulna	Wrist forearm
7K1LM00	Closed reduction of fracture of wrist	Wrist forearm
S23C.00	Fracture of lower end of both ulna and radius	Wrist forearm
S23..00	Fracture of radius and ulna	Wrist forearm
S234B00	Closed fracture radial styloid	Wrist forearm
7K1LL00	Closed reduction of fracture of radius and or ulna	Wrist forearm
S230700	Closed fracture radius, neck	Wrist forearm
S239.00	Fracture of shaft of radius	Wrist forearm
S242.00	Fracture at wrist and hand level	Wrist forearm
S238.00	Fracture of shaft of ulna	Wrist forearm
S293.00	Multiple fractures of forearm	Wrist forearm
S234300	Closed fracture of ulna, styloid process	Wrist forearm
S234F00	Closed Barton’s fracture	Wrist forearm
S23A.00	Fracture of shafts of both ulna and radius	Wrist forearm
S24..11	Hand fracture—carpal bone	Wrist forearm
S23..11	Forearm fracture	Wrist forearm
S234900	Closed volar Barton’s fracture	Wrist forearm
S240.00	Closed fracture of carpal bone	Wrist forearm
S23x.00	Closed fracture of radius and ulna, unspecified part	Wrist forearm
S240700	Closed fracture capitate	Wrist forearm
S230200	Closed fracture of ulna, coronoid	Wrist forearm
S4C2.00	Closed fracture-subluxation of the wrist	Wrist forearm
S4C0000	Closed fracture-dislocation distal radio-ulnar joint	Wrist forearm
S23 × 100	Closed fracture of radius (alone), unspecified	Wrist forearm
S234.00	Closed fracture of radius and ulna, lower end	Wrist forearm
S234000	Closed fracture of forearm, lower end, unspecified	Wrist forearm
S4C..00	Fracture-dislocation or subluxation of wrist	Wrist forearm
S234D00	Closed fracture distal radius, extra-articular, other type	Wrist forearm
S24..00	Fracture of carpal bone	Wrist forearm
S234211	Dupuytren’s fracture, radius—closed	Wrist forearm
S23 × 200	Closed fracture of ulna (alone), unspecified	Wrist forearm
S232.00	Closed fracture of radius and ulna, shaft	Wrist forearm
S234z00	Closed fracture of forearm, lower end, NOS	Wrist forearm
S4C0300	Closed fracture-dislocation, carpometacarpal joint	Wrist forearm
S234E00	Closed fracture distal radius, intra-articular, other type	Wrist forearm
S234600	Closed fracture radius and ulna, distal	Wrist forearm
S232z00	Closed fracture of radius and ulna, shaft, NOS	Wrist forearm
S230900	Closed fracture of the proximal radius	Wrist forearm
S240z00	Closed fracture of carpal bone NOS	Wrist forearm
S230000	Closed fracture of proximal forearm, unspecified part	Wrist forearm
S230800	Closed fracture proximal radius, comminuted	Wrist forearm
S230400	Closed fracture of proximal ulna, comminuted	Wrist forearm
S230500	Closed fracture of the proximal ulna	Wrist forearm
S4C0.00	Closed fracture dislocation of wrist	Wrist forearm
S232300	Closed fracture radius and ulna, middle	Wrist forearm
S23xz00	Closed fracture of radius and ulna, NOS	Wrist forearm
S4C0100	Closed fracture-dislocation radiocarpal joint	Wrist forearm
S234800	Closed Galeazzi fracture	Wrist forearm
S234500	Closed fracture distal ulna, unspecified	Wrist forearm
S234400	Closed fracture of ulna, lower epiphysis	Wrist forearm
S230z00	Closed fracture of proximal forearm not otherwise specified	Wrist forearm
S230.00	Closed fracture of proximal radius and ulna	Wrist forearm
S24z.00	Fracture of carpal bone NOS	Wrist forearm
S230A00	Closed fracture radius and ulna, proximal	Wrist forearm
S4C2000	Closed fracture-subluxation, distal radio-ulnar jt	Wrist forearm
S232200	Closed fracture of the ulnar shaft	Wrist forearm
S234C00	Closed fracture distal radius, intra-articular, die-punch	Wrist forearm
S234A00	Closed dorsal Barton’s fracture	Wrist forearm
S4C2100	Closed fracture-subluxation radiocarpal joint	Wrist forearm
S23 × 000	Closed fracture of forearm, unspecified	Wrist forearm
S232000	Closed fracture of radius, shaft, unspecified	Wrist forearm
S234111	Smith’s fracture—closed	Wrist forearm
Syu5400	[X]Fracture of forearm, unspecified	Wrist forearm
Syu6500	[X]Fracture of other & unspecified parts of wrist and hand	Wrist forearm
S234911	Closed volar Barton’s fracture-dislocation	Wrist forearm
S240y00	Closed fracture of other carpal bone	Wrist forearm
S234A11	Closed dorsal Barton’s fracture-dislocation	Wrist forearm
S240000	Closed fracture of carpal bone, unspecified	Wrist forearm
S4B0100	Closed fracture-dislocation superior radio-ulnar joint	Wrist forearm
S234912	Closed volar Barton fracture-subluxation	Wrist forearm
S4C0200	Closed fracture-dislocation mid carpal	Wrist forearm
S4C2300	Closed fracture-subluxation, carpometacarpal joint	Wrist forearm
S240F00	Closed fracture carpal bones, multiple	Wrist forearm
S4C2y00	Closed fracture-subluxation other carpal	Wrist forearm
Syu6300	[X]Fracture of other carpal bone(s)	Wrist forearm
S234G00	Greenstick fracture of distal radius	Wrist forearm
Syu5300	[X]Fracture of other parts of forearm	Wrist forearm
S4C2200	Closed fracture-subluxation mid carpal	Wrist forearm
S4B2100	Closed fracture-subluxation superior radio-ulnar joint	Wrist forearm
S234A12	Closed dorsal Barton fracture-subluxation	Wrist forearm
S235F00	Open Barton’s fracture	Wrist forearm open
S235100	Open Colles’ fracture	Wrist forearm open
S235211	Dupuytren’s fracture, radius—open	Wrist forearm open
S30..11	Hip fracture	Hip
S30..00	Fracture of neck of femur	Hip
S302.00	Closed fracture of proximal femur, pertrochanteric	Hip
7K1L400	Closed reduction of fracture of hip	Hip
S302400	Closed fracture of femur, intertrochanteric	Hip
7K1J000	Cls red + int fxn proximal femoral # + screw/nail device alone	Hip
7K1D01E	DHS—Dynamic hip screw primary fixation of neck of femur	Hip
S30y.11	Hip fracture NOS	Hip
7K1D01F	Dynamic hip screw primary fixation of neck of femur	Hip
S300500	Cls # prox femur, subcapital, Garden grade unspec	Hip
S30y.00	Closed fracture of neck of femur NOS	Hip
S302000	Cls # proximal femur, trochanteric section, unspecified	Hip
S302011	Closed fracture of femur, greater trochanter	Hip
S30w.00	Closed fracture of unspecified proximal femur	Hip
S304.00	Pertrochanteric fracture	Hip
S300700	Closed fracture proximal femur, subcapital, Garden grade II	Hip
S300900	Closed fracture proximal femur, subcapital, Garden grade IV	Hip
S300600	Closed fracture proximal femur, subcapital, Garden grade I	Hip
7K1J500	Primary int fxn(no red) prox fem # + screw/nail device alone	Hip
S300400	Closed fracture head of femur	Hip
S300800	Closed fracture proximal femur, subcapital, Garden grade III	Hip
S300.00	Closed fracture proximal femur, transcervical	Hip
7K1J700	Primary int fxn(no red) prox fem # + screw/nail + plate device	Hip
7K1Jd00	Closed reduction of intracapsular # NOF internal fixat DHS	Hip
S300000	Cls # prox femur, intracapsular section, unspecified	Hip
7K1J012	Cl red intracaps fract neck femur fix—Smith-Petersen nail	Hip
7K1J600	Primary int fxn(no red) prox fem # + scrw/nail + intramed device	Hip
S302z00	Cls # of proximal femur, pertrochanteric section, NOS	Hip
S302100	Closed fracture proximal femur, intertrochanteric, two part	Hip
S300A00	Closed fracture of femur, upper epiphysis	Hip
7K1JD00	Primary cls red + int fxn prox fem # + screw/nail + plate device	Hip
S302012	Closed fracture of femur, lesser trochanter	Hip
S300y00	Closed fracture proximal femur, other transcervical	Hip
S302300	Cls # proximal femur, intertrochanteric, comminuted	Hip
S300311	Closed fracture, base of neck of femur	Hip
S300300	Closed fracture proximal femur, basicervical	Hip
7K1J011	Cl red intracaps frac neck femur fix-Garden cannulated screw	Hip
7K1JC00	Prim cls rd + int fxn prox fem # + screw/nail + intramdulry device	Hip
7K1JB00	Primary cls red + int fxn prox fem # + screw/nail device alone	Hip
7K1J013	Cls red + int fxn prox femoral # + Richard’s cannulat hip screw	Hip
S300z00	Closed fracture proximal femur, transcervical, NOS	Hip
S300200	Closed fracture proximal femur, midcervical section	Hip
S300y11	Closed fracture of femur, subcapital	Hip
S300100	Closed fracture proximal femur, transepiphyseal	Hip
7K1K300	Primary external fixation(without reduction) prox femoral #	Hip
7K1K500	Primary cls reduction + external fixation proximal femoral #	Hip
7K1Y000	Remanip intracap fract neck fem and fix using nail or screw	Hip
7K1D000	Prmy open red + int fxn prox femoral # + screw/nail + plate device	Hip open
S301800	Open fracture proximal femur, subcapital, Garden grade III	Hip open
7K1D01A	Prim open reduct # neck femur & op fix—Richards screw	Hip open
7K1D600	Prmy open red + int fxn prox femoral # + screw/nail device alone	Hip open
7K1D700	Prmy open red + int fxn prox fem # + screw/nail + intramed device	Hip open
S30z.00	Open fracture of neck of femur NOS	Hip open
S301500	Open fracture proximal femur,subcapital, Garden grade unspec	Hip open
S303400	Open fracture of femur, intertrochanteric	Hip open
7K1G200	Primary open reduction + external fixation of femoral fracture	Hip open
7K1D018	Prim open reduct # neck femur & op fix—Neufield nail plate	Hip open
S301000	Opn # proximal femur, intracapsular section, unspecified	Hip open
S301900	Open fracture proximal femur,subcapital, Garden grade IV	Hip open
7K1D012	Prim op red # nck femur & op fix- Charnley compression screw	Hip open
7K1D017	Prim open red # neck femur & op fix—McLaughlin nail plate	Hip open
7K1D01B	Prim open reduct # neck femur & op fix—Ross Brown nail	Hip open
7K1D01D	Prim op red # nck femur & op fix- Zickel intramed nail plate	Hip open
S30x.00	Open fracture of unspecified proximal femur	Hip open
7K1D011	Prim open reduct # neck femur & op fix—Blount nail plate	Hip open
S301600	Open fracture proximal femur,subcapital, Garden grade I	Hip open
S303.00	Open fracture of proximal femur, pertrochanteric	Hip open
7K1D019	Prim open reduct # neck femur & op fix—Pugh nail plate	Hip open
S301700	Open fracture proximal femur,subcapital, Garden grade II	Hip open
S303000	Open # of proximal femur, trochanteric section, unspecified	Hip open
S301y00	Open fracture proximal femur, other transcervical	Hip open
S303z00	Open fracture of proximal femur, pertrochanteric, NOS	Hip open
S301100	Open fracture proximal femur, transepiphyseal	Hip open
S301y11	Open fracture of femur, subcapital	Hip open
S301.00	Open fracture proximal femur, transcervical	Hip open
7K1D014	Prim open reduct # neck femur & op fix—Holt nail	Hip open
S301A00	Open fracture of femur, upper epiphysis	Hip open
S303011	Open fracture of femur, greater trochanter	Hip open
7K1D013	Prim op red # nck femur & op fix—Deyerle multiple hip pin	Hip open
S303300	Open fracture proximal femur, intertrochanteric, comminuted	Hip open
S301311	Open fracture base of neck of femur	Hip open
S303100	Open fracture proximal femur, intertrochanteric, two part	Hip open
7K1D015	Prim open reduct # neck femur & op fix—Jewett nail plate	Hip open
7K1DE00	Prim op red frac neck fem op fix us prox fem nail antirotatn	Hip open
7K1D016	Prim open reduct # neck femur & op fix—Massie nail plate	Hip open
